# X-ray dark-field tomography reveals tooth cracks

**DOI:** 10.1038/s41598-021-93393-4

**Published:** 2021-07-07

**Authors:** Christoph Jud, Yash Sharma, Benedikt Günther, Jochen Weitz, Franz Pfeiffer, Daniela Pfeiffer

**Affiliations:** 1 Chair of Biomedical Physics, Department of Physics and Munich School of BioEngineering, Technical University of Munich, 85748 Garching, Germany; 2 Max-Planck Institute of Quantum Optics, 85748 Garching, Germany; 3 Department of Oral and Maxillofacial Surgery, Klinikum rechts der Isar, Technical University of Munich, 81675 Munich, Germany; 4 Department of Diagnostic and Interventional Radiology, Klinikum rechts der Isar, Technical University of Munich, 81675 Munich, Germany

**Keywords:** Medical imaging, Applied physics

## Abstract

Cracked tooth syndrome (CTS) is a common clinical finding for teeth, it affects about 5% of all adults each year. The finding of CTS is favored by several risk factors such as restorations, bruxism, occlusion habits, and age. Treatment options range, depending on the severity, from no treatment at all to tooth extraction. Early diagnosis of CTS is crucial for optimal treatment and symptom reduction. There is no standard procedure for an evidence-based diagnosis up to date. The diagnosis is a challenge by the fact that the symptoms, including pain and sensitivity to temperature stimuli, cannot be clearly linked to the disease. Commonly used visual inspection does not provide in-depth information and is limited by the resolution of human eyes. This can be overcome by magnifying optics or contrast enhancers, but the diagnosis will still strongly rely on the practicians experience. Other methods are symptom reproduction with percussions, thermal pulp tests or bite tests. Dental X-ray radiography, as well as computed tomography, rarely detect cracks as they are limited in resolution. Here, we investigate X-ray dark-field tomography (XDT) for the detection of tooth microcracks. XDT simultaneously detects X-ray small-angle scattering (SAXS) in addition to the attenuation, whereas it is most sensitive to the micrometer regime. Since SAXS originates from gradients in electron density, the signal is sensitive to the sample morphology. Microcracks create manifold interfaces which lead to a strong signal. Therefore, it is possible to detect structural changes originating from subpixel-sized structures without directly resolving them. Together with complementary attenuation information, which visualizes comparatively large cracks, cracks are detected on all length-scales for a whole tooth in a non-destructive way. Hence, this proof-of principle study on three ex-vivo teeth shows the potential of X-ray scattering for evidence-based detection of cracked teeth.

## Introduction

Cracked tooth syndrome (CTS) affects about 5% of all adults each year and is one of the most common clinical findings for teeth^1^. In about 15% of all cases, the crack either affects the pulp or leads to tooth extraction^2^. CTS is most common in mandibular molars^3,4^, whereas about 65% of all cracks are found in teeth with restorations^5^, since the amount of supporting tooth structure is reduced and may no longer withstand masticatory forces^6–8^. However, tooth crowns are used to prevent cracks as well, which was found by a study in North Carolina^2^.

Apart from iatrogenic causes such as restorations, natural risk factors include bruxism, occlusion habits, extensive attrition and abrasion^9,10^. Older patients over 40 years are more affected by CTS, making it a problematic side-effect of a growing average lifespan^3,7,8,11^.

The symptoms can include pain, bite problems and sensitivity to temperature stimuli^12,13^. It is not ideal to use them as the sole source of information since they are not uniquely identified with CTS. Despite the vast number of cases, however, there is still no standard procedure for an evidence-based diagnostic tool to confirm CTS. A common diagnostic procedure is a visual inspection, which does not yield any in-depth information. Although this technique is also limited by the resolution of human eyes of about 200 µm^14^, microscopic examination and contrast enhancers such as methylene blue dye can overcome this challenge. In the transillumination method, a fiber optic is used to illuminate the tooth. If some crack extends to the dentin, the light gets distorted which can be inspected using a magnifying optic^7,15^. To reproduce the symptoms, percussions, thermal pulp tests or bite-tests can be performed, whereas pain upon release is an indicator for cracks^10^. Other alternatives for the diagnosis of CTS are ultrasound testing^16^, infrared thermography^17^, or optical coherence tomographic imaging^18,19^. X-ray radiography is routinely used to diagnose the pulp health and the gums but rarely detects cracks^20^. Cone-beam computed tomography provides additional 3D-information but has a limited spatial resolution^21^. Even high-resolution micro-CTs can typically identify cracks only if they are larger than 80 µm. However, the early detection of microcracks is crucial to prevent secondary infections and further crack propagation^5^.

Over the last decade, grating-based phase-contrast X-ray imaging has been transferred from synchrotron facilities to laboratory X-ray sources^23^. Grating-based X-ray imaging thereby allows measuring the refractive index as well as small-angle X-ray scattering (SAXS) in addition to the conventional attenuation signal^23–25^. This is done by a so-called Talbot-Lau-interferometer, i.e., a particular geometrical arrangement of three optical gratings, which converts phase information and scattering properties of the specimen under investigation into a measurable intensity signal^23,25^. The SAXS-signal is ultimately caused by gradients in electron density. Thus, it is related to the sample morphology^26^. In a Talbot–Lau-interferometer, structures are mostly detected if they are in the same order of magnitude as the setup autocorrelation length, which is in the micrometer regime for typical setup designs^27,28^. Moreover, a scattering signal is predominantly detected in one distinct sensitivity direction^29^. This can be exploited by sample rotation around the optical axis to retrieve directional-dependent scattering information in projection geometry^30–33^. Multiple projections for a complete set of sample orientations allow the reconstruction of three-dimensional scattering tensors, known as anisotropic X-ray dark-field tomography (anisotropic XDT)^34,35^. It has been shown that the signal correlates well with the orientation of fibrous structures and can be used to detect the orientation of dentinal tubules^36^.

Here, we evaluate the XDT as a tool for the detection of tooth microcracks. The complementary information provided by X-ray scattering is investigated and compared to the simultaneously acquired attenuation signal. Since XDT detects a mean signal originating from subpixel sized structures, we can indirectly detect microstructure features for a whole tooth in a non-destructive measurement. Hence, this method could be used to identify tooth cracks in an early stage, potentially improving both the sensitivity and specificity of CTS-diagnosis.

## Materials and methods

Please note that parts of this section are already described in Ref.^37^. The experiment was approved by the local ethics committee (Ethics Committee of the Technical University of Munich), written informed consent was waived for this retrospective analysis. All methods were carried out in accordance with relevant guidelines and regulations. Three human teeth (upper left third molar, upper first molar, second premolar) were extracted from different donors and immediately conserved in a buffer solution.

### Sample preparation

To conserve the samples, they were embedded in an Araldite (Huntsman corp., Salt Lake City, USA) epoxy resin. Hence, the specimens were gradually dehydrated to a 100% acetone solution. The used concentrations (all vol/vol) for the dehydration series were in %: 30, 50, 70, 80, 90, 95 and 100 acetone balanced with distilled water. The dehydration incubations were performed for 1 h each. Subsequently, the sample was embedded in Araldite A, i.e. a mixture of Araldite M and Hardener in a weight ratio of 1:1. This process was repeated in a ratio of Araldite A and Acetone of 1:3 for 1 h, 1:1 for 4 h, 3:1 for 2 h and 100% Araldite A overnight. Finally, the samples were embedded in Araldite B (10 g Araldite M, 10 g Hardener and 0.6 g Accelerator) and heated at 60 °C for two days.

### Setup parameter

**Figure 1:**
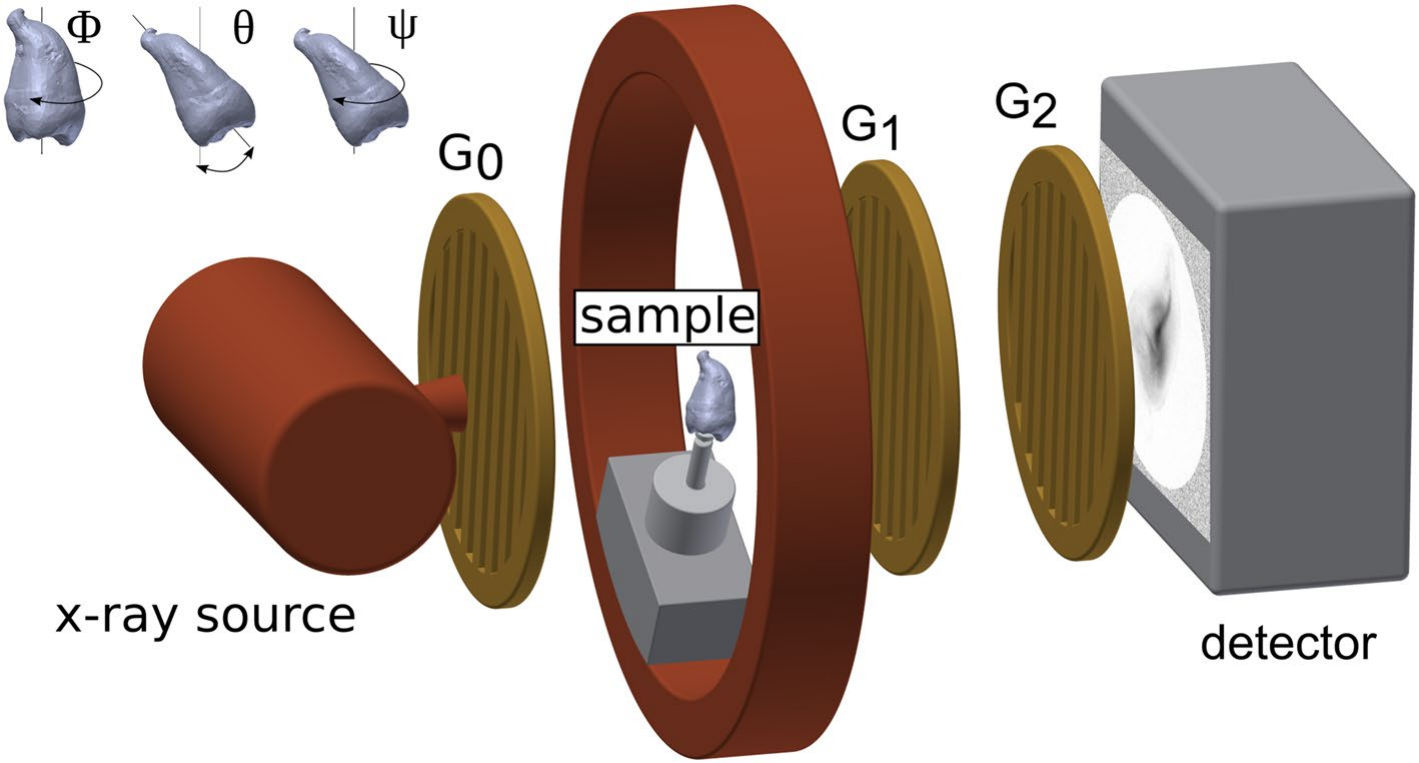
Schematic overview of the experimental setup, including a grating interferometer. The source grating G0 provides enough spatial coherence to generate a self-interference effect after the phase-grating G1. The subsequent modulation is recorded by a phase-stepping procedure with the attenuation grating G2 and yields the attenuation as well as information about the refractive index and small-angle X-ray scattering.

The experimental setup illustrated in Fig. 1 consisted of a microfocus X-ray tube (X-ray WorX XWT-160-SE) and a flat panel detector (Varian 2520DX) with a pixel pitch of 127 µm. The tube voltage was 60 kV with a power of 20 W (i.e. 0.33 mA). A Talbot–Lau grating interferometer with a design energy of 45 keV allowed to measure dark-field images in addition to the conventional attenuation. As sketched in Fig. 1, the interferometer consists of two attenuation gratings (G_0_ and G_2_) with periods of 10 µm and a phase-shifting grating (G_1_) with a period of 5 µm. The phase-grating consisted of Ni with a height of 8 µm which phase-shifts X-rays at the design energy by a factor of π/2. The gratings were symmetrically adjusted with around the G_1_ with an inter-grating distance of 92.7 cm, more details about the grating interferometer can be found in Prade et al.^28^. A single dark-field image was measured with a phase-stepping procedure consisting of 5 single acquisitions at different positions of phase-grating G_1_ with an exposure time of 5 s each.

### X-ray dark-field tomography

The sample was mounted in an Eulerian cradle which allowed almost free sample rotation in space according to the Eulerian angles ψ, θ, and φ as depicted in Fig. 1. To cover a large range of sensitivity directions, 1025 sample projections were measured in total. With a measurement time of 25 s per projection, this resulted in a total measurement time of about 7 h. Their orientations were distributed following an optimized acquisition scheme which covers a maximum of different scattering directions in the dark-field contrast modality^38^. The XDT was then reconstructed with to an algorithm developed by^35^. From the reconstruction results, we used the mean scattering strength as well as the attenuation in our analysis.

## Results

**Figure 2:**
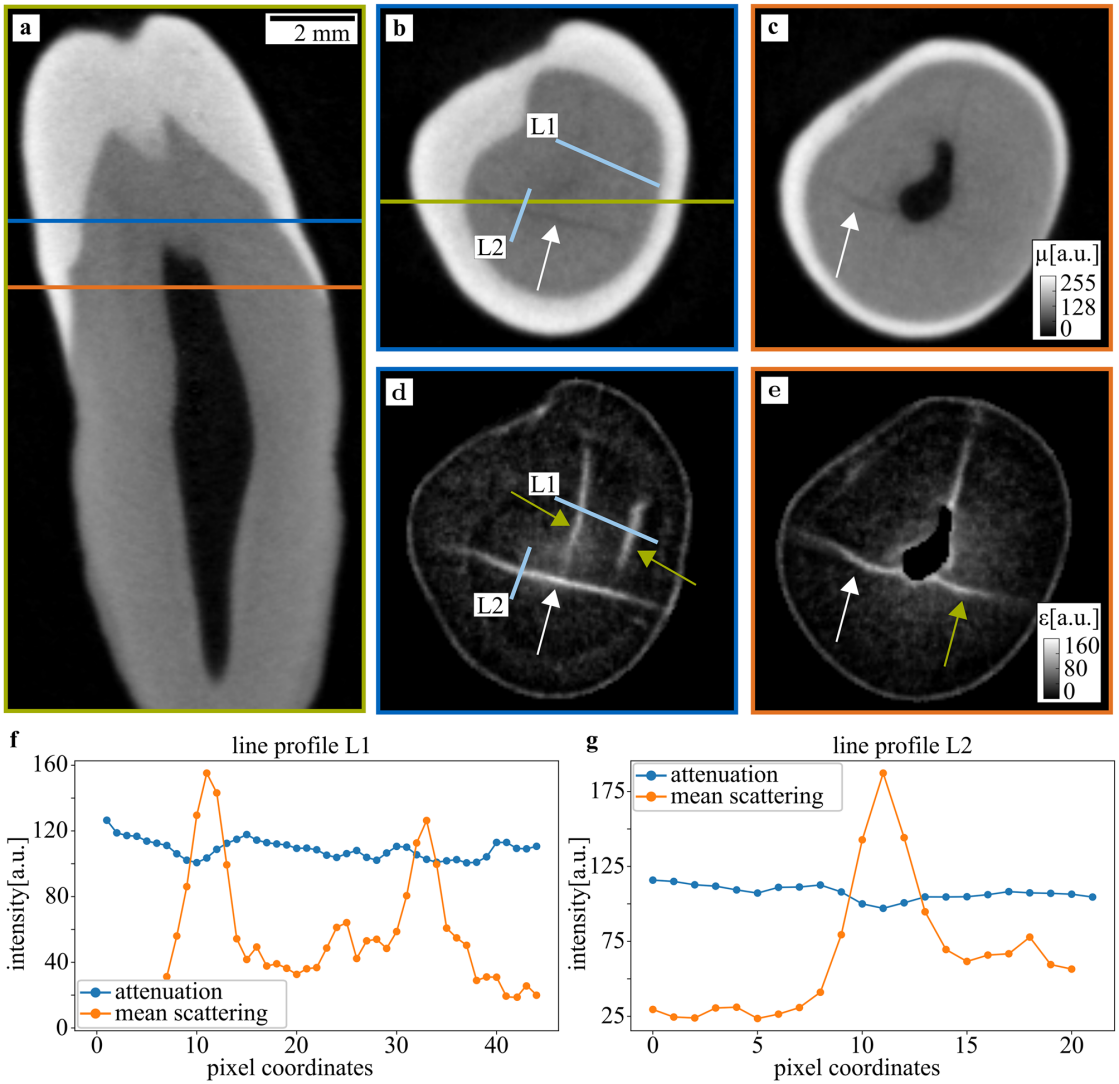
Attenuation and mean scattering tomographic images of a molar tooth. In (**a**), a sagittal slice through the reconstruction depicts enamel, dentin, and pulp, which are also visible in the axial slices in (**b**) and (**c**). The dentin region is homogeneous, a crack is indicated by a white arrow. Additionally, an inlet shows the line-plot L3 with the attenuation (blue) and scattering strength (orange). The corresponding scattering signal is illustrated in (**d**) and (**e**) and shows the same crack (white arrow) as well as additional ones indicated by green arrows. Two line-plots L1 and L2 highlight the difference between both contrast modalities. The slice positions are indicated by colored lines, a scale bar indicates the approximate sample size.

The first sample was an upper left third molar tooth of an adult male. In Fig. 2, slices through both the attenuation reconstruction (a–c) and the mean scattering (d, e) are shown. Both contrast modalities are simultaneously reconstructed from identical raw data, and hence perfectly registered to each other. The slice positions are indicated by colored lines, the data was windowed for maximal contrast. In the attenuation image, the main tooth regions such as enamel, dentin and pulp chamber can be easily distinguished. Cracks are visible in the axial slices, their position is indicated by white arrows. In contrast to the attenuation, there is little contrast between enamel and dentin in the mean scattering image, the pulp chamber has been masked using the attenuation data. However, the cracks barely visible in the attenuation image are now the most prominent image feature. Moreover, additional cracks are indicated by green arrows. To get a quantitative comparison between both contrast modalities, two line-plots L1 and L2 are presented in (f) and (g), their position is indicated in the corresponding axial slices. The attenuation shown in blue is quite homogeneous, only a small intensity loss indicates the crack positions. In the mean scattering, however, crack positions can be easily identified. Quantitatively, we define the visibility *V* as

1 $$V = \frac{{I_{{max}} - I_{{min}} }}{{I_{{max}} + I_{{min}} }},$$

where *I*_*max*_ and *I*_*min*_ correspond to the maximal and minimal intensity along the line-plot. With this definition, a peak with a low background level of zero leads to high visibility approaching 100% whereas a homogeneous intensity distribution yields low visibility. The line-plot L1 yields low visibility of 11% for the attenuation compared to 93% for the scattering signal. For line-plot L2, the attenuation has visibility of 9% and the scattering visibility of 78%.

**Figure 3:**
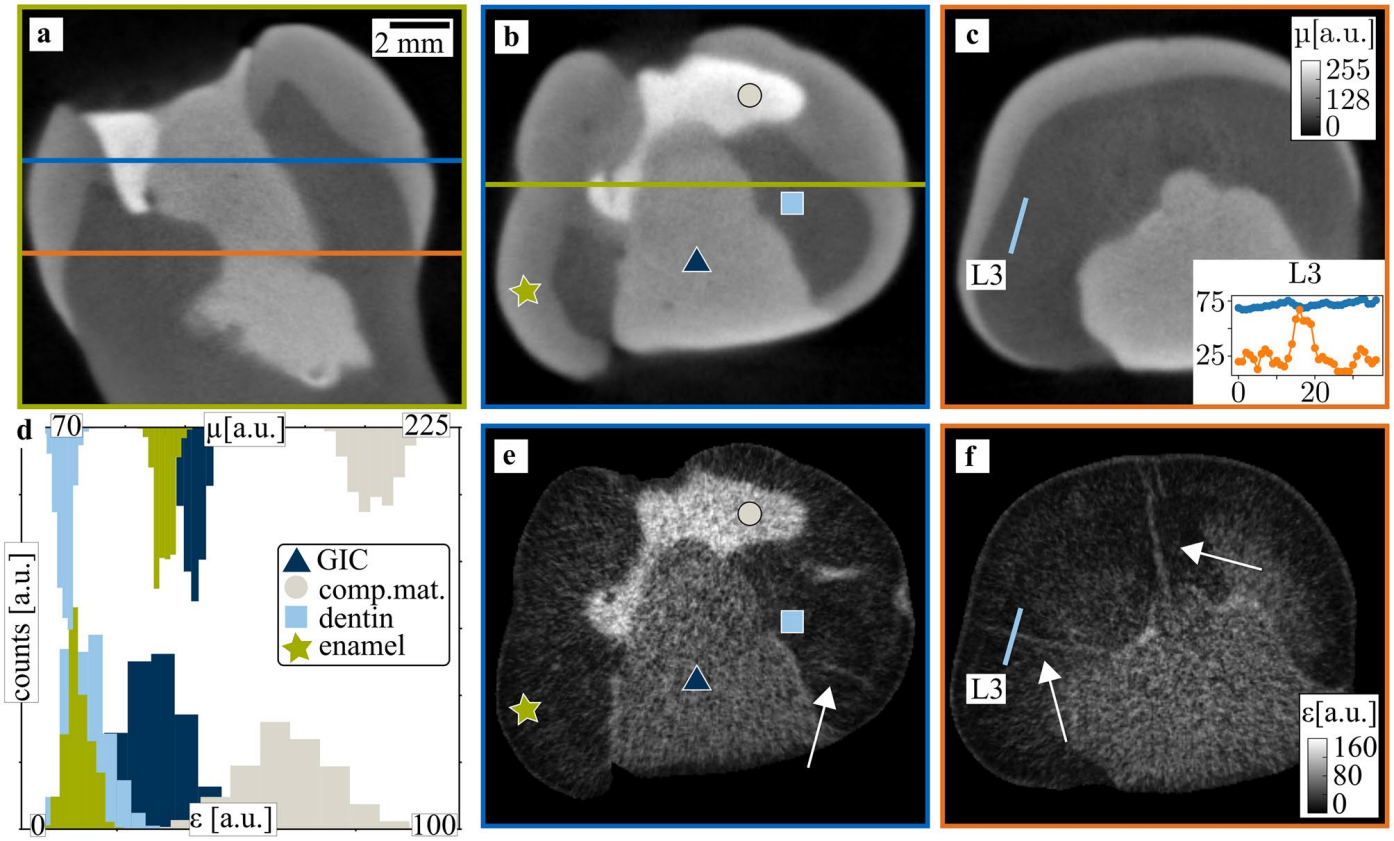
Attenuation and mean scattering tomography images for a tooth with fillings. A sagittal slice (**a**) and two axial slices (**b**,**c**) illustrate the attenuation signal. The inlet in (**c**) depicts the line-plot L4 with the attenuation (blue) and scattering strength (orange). In (**d**), histograms illustrate the attenuation and mean scattering strength for different tooth regions. The composite material filling and the glass ionomer cement (GIC) are depicted as well. In (**e**,**f**), the mean scattering signal corresponding to the attenuation slices is shown. Some cracks are visible in this contrast modality and are oblique in the attenuation signal, as indicated by white arrows.

Figure 3 illustrates the results obtained with the second sample, an upper first molar, with two different fillings which are visible in the attenuation slices (a–c) as bright regions. One consists of composite material as marked by a gray circle, it covers a part of the tooth crown. The other filling is composed of glass ionomer cement (GIC) as marked by a dark-blue triangle, it extends all the way to the tooth root. In addition, dentin and enamel are marked by a blue square and a green star. In Fig. 3d, histograms of the grayscale values within a region of interest around the markers are shown, their size is approximately equal to the size of the markers. A gaussian fit to the histogram of each region of interestyields the mean values and standard deviation for different tooth regions, the values are given in Table 1. The composite material filling attenuates most with a mean value of µ = 204 ± 9, GIC has a lower attenuation value of µ = 129 ± 5, which is close to the value of enamel (µ = 116 ± 4). In contrast to the attenuation, their mean scattering values are quite different: GIC has a mean scattering strength of ɛ = 29 ± 8, whereas enamel has ɛ = 10 ± 3. However, the mean scattering strengths for enamel and dentin are very close, which results in overlapping histograms for those tooth regions. In Fig. 3e,f, three cracks are indicated by white arrows. They spread radially from GIC towards the surface. The inlet in Fig. 3c) depicts the visibility along line-plot L3. The pixel index is plotted versus the attenuation (blue) or scattering strength (orange), both in arbitrary units. The attenuation yielded a visibility of 7%, compared to 72% for the scattering.

**Table 1:** Attenuation and mean scattering strength for different tooth regions. The values are calculated from a Gaussian fit to the histogram of a region of interest in the respective tooth region.

Tooth region	Attenuation (a.u.)	Mean scattering (a.u.)
Composite material	204 ± 9	63 ± 12
Glass ionomer cement	129 ± 5	29 ± 8
Dentin	75 ± 3	12 ± 5
Enamel	116 ± 4	10 ± 3

**Figure 4:**
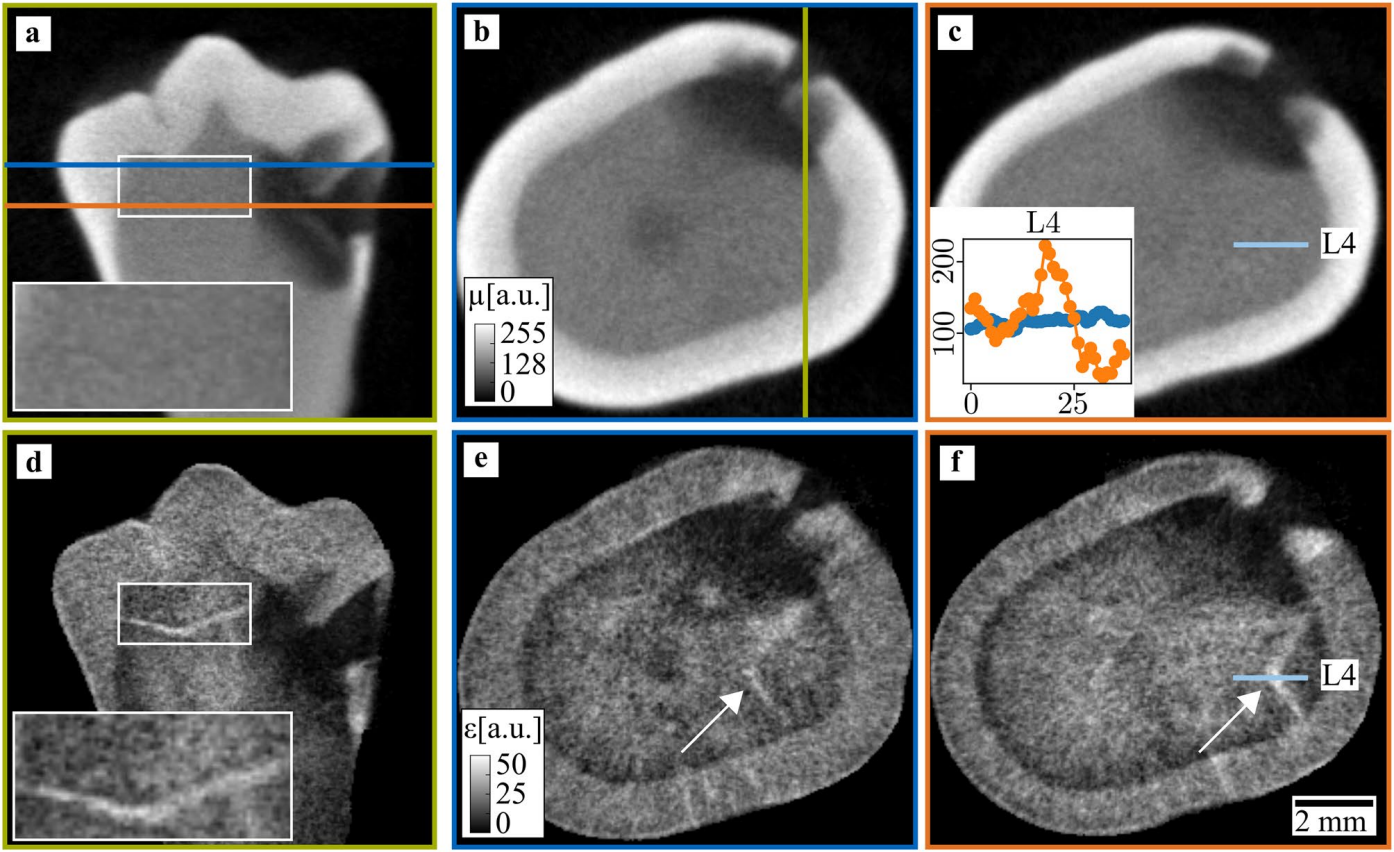
Attenuation and mean scattering tomography images of a carious tooth. In the first row, a sagittal slice (**a**) and two axial slices (**b**,**c**) show a cavity in the enamel as well as the carious region within the dentin. The scattering signal (**d**,**e**) allows to identify those regions as well and additionally detects a crack (magnified region and white arrows). In the attenuation images, however, the crack is not visible.

The third sample was a second premolar and is depicted in Fig. 4. It had a carious infection, which caused a smooth surface cavity, as can be seen in the attenuation slices in Fig. 4a–c), their positions are again indicated by colored lines. The disease already penetrated the dentin region but did not spread to the pulp yet. In the second row (Fig. 4d–f), the corresponding mean scattering slices detect the cavity and the affected dentin region as well. A decrease in scattering can be seen close to the enamel-dentin border, allowing them to be distinguished in the mean scattering signal. Additionally, a crack is visible extending from the enamel to the diseased tooth region. A closeup look allows a direct comparison between mean scattering signal and the attenuation, where the crack is not detected. Quantitatively, this can be seen by a line plot depicted in Fig. 4c). Again, the horizontal axis depicts the pixel coordinate, and the vertical axis attenuation (blue) and scattering strength (orange), respectively. The visibility for the attenuation is 11%, for the scattering strength it is 70%.

## Discussion

Although CTS is very common, there is no generally accepted and concise diagnostic tool available. Currently applied methods often rely on the reproduction of symptoms, which leaves much space for ambiguities and interpretation by the practicians.

Other imaging tools such as X-ray radiography rely on attenuation as contrast source which in turn strongly depends on the atomic number as well as the material density. Even though X-rays easily penetrate teeth, radiographs only detect the average attenuation coefficient along the beam direction while losing all in-depth information. More detailed information is retrieved by three-dimensional methods such as cone-beam computed tomography (CBCT) or XDT, which was used in this *ex-vivo* study. As can be seen in Fig. 2, the attenuation reconstruction allows distinguishing between enamel, dentin and the pulp chamber due to their different density and material composition.

Tooth cracks, however, don’t change the natural tooth structure but only cause a translocation. In projection geometry, such changes are only visible if the view is well aligned to the crack direction, which cannot be assumed in general. In three-dimensional reconstructions, the crack detection depends upon the spatial resolution, which is limited to about 80 µm for some imaging systems^22^. In Fig. 2, the reconstruction had a voxel size of (40 ± 3) µm, giving a lower limit to the spatial resolution. This allowed identifying several tooth cracks such as the one indicated by a white arrow in Fig. 2b). The corresponding line-plot L2 shows a decrease in the attenuation signal, which is evident since the density decreases in the crack region. However, it is impossible to detect microcracks with a size smaller than the resolution such as the microcracks indicated by green arrows.

XDT measures the mean scattering signal in addition to the attenuation, which is sensitive to gradients in electron density. As those gradients are especially high at boundaries between different materials, the mean scattering signal is well suited to detect morphological sample changes instead of material composition. Hence, the signal in a single voxel represents the mean isotropic scattering averaged over the spatial resolution. The origin of the scattering signal, however, may be structures that can be one order of magnitude smaller than the spatial resolution. The signal is thereby most sensitive to features comparable to the setup autocorrelation length, which is given by setup-specific parameters and in this study was lower than 2.5 µm^28^. This enables detection of microscopical structure changes including microcracks without directly resolving them.

Since the morphology rapidly changes in a crack region, a strong scattering signal is detected for cracks on top of a relatively low background, as visible in Fig. 2, where the mean scattering signal allows detecting additional microcracks with respect to the attenuation signal. This explains the difference between both contrast modalities in the line-plots in Fig. 2f,g), where the scattering signal has much higher visibilities of 78% and 93% compared to the attenuation signal with visibilities of 9% and 11%. If compromises regarding spatial resolution have to be made due to the increased specimen's size, no microcracks are detected at all in the attenuation signal. Nevertheless, they remain still visible in the mean scattering signal. This is especially well depicted in Fig. 4a,d, where a region of interest shows a close-up view of the tooth crack.

Figure 3 shows a typical tooth structure resulting from a root canal treatment (RCT). Part of the tooth crown is covered with composite material, the rest has been filled with glass ionomer cement (GIC). Its attenuation of µ = 129 ± 5 is quite similar to the attenuation of enamel (µ = 116 ± 4). However, they are easily distinguishable in the mean scattering strength contrast modality with a higher value of ɛ = 29 ± 8 for GIC compared to a value of ɛ = 10 ± 3 for enamel. For other materials such as enamel and dentin, the best contrast is provided by the attenuation signal whereas the mean scattering provides no contrast, highlighting the complementarity of both contrast modalities.

The sample presented in Fig. 4 suffered from dental caries, which caused a smooth surface cavity. As the disease progresses in the enamel, the surface region remains relatively well-mineralized^39^. This can be seen in Fig. 4b,c, where the enamel surface has a higher attenuation coefficient close to the surface compared to the inner part. In the mean scattering signal, there is no difference between the surface and the inner part, which suggests that demineralization does not lead to an increased scattering signal.

In the dentin region, the caries infection progresses through the dentinal tubules towards the pulp chamber, leading to the triangular shape visible in Fig. 4. In contrast to the enamel, dentin reacts to dental caries infection by the formation of sclerotic dentin. This reduces the size of the dentinal tubules with mineral materials from odontoblasts, which reduces the number of interfaces in the dentin and ultimately leads to a decreased scattering signal.

In contrast to symptom reproducers, XDT provides in-depth information and does not rely on the subjective interpretation of a practitioner. Moreover, it can detect both large-scale cracks in the simultaneously reconstructed attenuation signal and microcracks in the scattering signal. Thermography and transillumination methods are capable to detect microscopic cracks as well, but they do not provide three-dimensional information. Hence, they might miss cracks in certain cases. In addition, thermography relies upon the heat production due to friction within microcracks, which makes it difficult to detect larger cracks which in turn would require an additional imaging method^17^.

However, several limitations must be overcome in order to apply XDT in clinical practice. Until now, the reconstruction algorithm requires sample projections from many different orientations, leading to an elaborate acquisition scheme and long acquisition times of several hours. In a future in-vivo application, the possible sample orientations would be limited which could be compensated by using prior knowledge about the sample. Moreover, the dose of this proof of principle study is not yet compatible with clinical dose levels. Dose-compatible measurements have been made already for other applications such as mammography or in-vivo lung imaging of pigs^40,41^.

In conclusion, we provided evidence in this *ex-vivo* study that XDT potentially is a powerful tool for the diagnosis of CTS. The simultaneous acquisition of both attenuation and scattering information provides complementary information, highlighting both the overall tooth structure and its morphology. Thereby, the mean scattering signal senses signal originating from structures one order of magnitude smaller than the imaging system resolution. Thus, it can detect tooth microcracks which are oblique in the attenuation signal.
